# Supplementary material for: Pyruvate Dehydrogenase Kinase 1 inhibition mediated oxidative phosphorylation enhancement in cartilage promotes osteoarthritis progression
Source: BMC Musculoskelet Disord. 2023 Jul 20;24:597. doi: 10.1186/s12891-023-06585-6 (PMC10357736; doi:10.1186/s12891-023-06585-6)
Supplement: Supplementary file 1 — Additional file 1. [file 12891_2023_6585_MOESM1_ESM.zip › BP.Gsea.1653623667859/GOBP_RIBONUCLEOSIDE_TRIPHOSPHATE_BIOSYNTHETIC_PROCESS.html]

Details for gene set GOBP\_RIBONUCLEOSIDE\_TRIPHOSPHATE\_BIOSYNTHETIC\_PROCESS[GSEA]

|  || Dataset | 55457\_collapsed\_to\_symbols.cls.cls #OA\_versus\_normal.cls.cls #OA\_versus\_normal\_repos |
| Phenotype | cls.cls#OA\_versus\_normal\_repos |
| Upregulated in class | OA |
| GeneSet | GOBP\_RIBONUCLEOSIDE\_TRIPHOSPHATE\_BIOSYNTHETIC\_PROCESS |
| Enrichment Score (ES) | 0.37289307 |
| Normalized Enrichment Score (NES) | 1.5628543 |
| Nominal p-value | 0.005063291 |
| FDR q-value | 0.28690434 |
| FWER p-Value | 1.0 |
Table: GSEA Results Summary

  

Fig 1: Enrichment plot: GOBP\_RIBONUCLEOSIDE\_TRIPHOSPHATE\_BIOSYNTHETIC\_PROCESS      
 Profile of the Running ES Score & Positions of GeneSet Members on the Rank Ordered List

  

| SYMBOL | TITLE | RANK IN GENE LIST | RANK METRIC SCORE | RUNNING ES | CORE ENRICHMENT || 1 | DGUOK | deoxyguanosine kinase [Source:HGNC Symbol;Acc:HGNC:2858] | 96 | 0.917 | 0.0375 | Yes |
| 2 | NME7 | NME/NM23 family member 7 [Source:HGNC Symbol;Acc:HGNC:20461] | 178 | 0.835 | 0.0721 | Yes |
| 3 | TREM2 | triggering receptor expressed on myeloid cells 2 [Source:HGNC Symbol;Acc:HGNC:17761] | 342 | 0.726 | 0.0949 | Yes |
| 4 | NME5 | NME/NM23 family member 5 [Source:HGNC Symbol;Acc:HGNC:7853] | 362 | 0.720 | 0.1287 | Yes |
| 5 | ATP5MC1 | ATP synthase membrane subunit c locus 1 [Source:HGNC Symbol;Acc:HGNC:841] | 465 | 0.674 | 0.1538 | Yes |
| 6 | ATP5PD | ATP synthase peripheral stalk subunit d [Source:HGNC Symbol;Acc:HGNC:845] | 564 | 0.635 | 0.1773 | Yes |
| 7 | ANTKMT | adenine nucleotide translocase lysine methyltransferase [Source:HGNC Symbol;Acc:HGNC:14152] | 618 | 0.618 | 0.2035 | Yes |
| 8 | NME4 | NME/NM23 nucleoside diphosphate kinase 4 [Source:HGNC Symbol;Acc:HGNC:7852] | 677 | 0.599 | 0.2283 | Yes |
| 9 | DMAC2L | distal membrane arm assembly component 2 like [Source:HGNC Symbol;Acc:HGNC:18799] | 695 | 0.595 | 0.2561 | Yes |
| 10 | ATP5PO | ATP synthase peripheral stalk subunit OSCP [Source:HGNC Symbol;Acc:HGNC:850] | 840 | 0.558 | 0.2722 | Yes |
| 11 | NME3 | NME/NM23 nucleoside diphosphate kinase 3 [Source:HGNC Symbol;Acc:HGNC:7851] | 876 | 0.549 | 0.2964 | Yes |
| 12 | ATP5PB | ATP synthase peripheral stalk-membrane subunit b [Source:HGNC Symbol;Acc:HGNC:840] | 1293 | 0.459 | 0.2865 | Yes |
| 13 | ATP5MF | ATP synthase membrane subunit f [Source:HGNC Symbol;Acc:HGNC:848] | 1330 | 0.453 | 0.3059 | Yes |
| 14 | NME2 | NME/NM23 nucleoside diphosphate kinase 2 [Source:HGNC Symbol;Acc:HGNC:7850] | 1428 | 0.439 | 0.3198 | Yes |
| 15 | STOML2 | stomatin like 2 [Source:HGNC Symbol;Acc:HGNC:14559] | 1819 | 0.382 | 0.3082 | Yes |
| 16 | PINK1 | PTEN induced kinase 1 [Source:HGNC Symbol;Acc:HGNC:14581] | 2097 | 0.350 | 0.3037 | Yes |
| 17 | ATP5F1C | ATP synthase F1 subunit gamma [Source:HGNC Symbol;Acc:HGNC:833] | 2190 | 0.339 | 0.3132 | Yes |
| 18 | ATP5MC2 | ATP synthase membrane subunit c locus 2 [Source:HGNC Symbol;Acc:HGNC:842] | 2371 | 0.318 | 0.3148 | Yes |
| 19 | SPHK2 | sphingosine kinase 2 [Source:HGNC Symbol;Acc:HGNC:18859] | 2478 | 0.305 | 0.3214 | Yes |
| 20 | TGFB1 | transforming growth factor beta 1 [Source:HGNC Symbol;Acc:HGNC:11766] | 2490 | 0.303 | 0.3355 | Yes |
| 21 | ATP5MC3 | ATP synthase membrane subunit c locus 3 [Source:HGNC Symbol;Acc:HGNC:843] | 2643 | 0.286 | 0.3376 | Yes |
| 22 | ATP5F1D | ATP synthase F1 subunit delta [Source:HGNC Symbol;Acc:HGNC:837] | 2701 | 0.279 | 0.3469 | Yes |
| 23 | ATP6V0C | ATPase H+ transporting V0 subunit c [Source:HGNC Symbol;Acc:HGNC:855] | 2790 | 0.271 | 0.3533 | Yes |
| 24 | IMPDH2 | inosine monophosphate dehydrogenase 2 [Source:HGNC Symbol;Acc:HGNC:6053] | 2867 | 0.264 | 0.3603 | Yes |
| 25 | ATP5MG | ATP synthase membrane subunit g [Source:HGNC Symbol;Acc:HGNC:14247] | 2941 | 0.256 | 0.3672 | Yes |
| 26 | ATP5F1B | ATP synthase F1 subunit beta [Source:HGNC Symbol;Acc:HGNC:830] | 3024 | 0.247 | 0.3729 | Yes |
| 27 | PID1 | phosphotyrosine interaction domain containing 1 [Source:HGNC Symbol;Acc:HGNC:26084] | 3280 | 0.222 | 0.3639 | No |
| 28 | PARP1 | poly(ADP-ribose) polymerase 1 [Source:HGNC Symbol;Acc:HGNC:270] | 3410 | 0.210 | 0.3642 | No |
| 29 | VCP | valosin containing protein [Source:HGNC Symbol;Acc:HGNC:12666] | 3518 | 0.201 | 0.3657 | No |
| 30 | ATP5F1A | ATP synthase F1 subunit alpha [Source:HGNC Symbol;Acc:HGNC:823] | 4137 | 0.143 | 0.3245 | No |
| 31 | NME6 | NME/NM23 nucleoside diphosphate kinase 6 [Source:HGNC Symbol;Acc:HGNC:20567] | 4257 | 0.132 | 0.3217 | No |
| 32 | ALDOA | "aldolase, fructose-bisphosphate A [Source:HGNC Symbol;Acc:HGNC:414]" | 4289 | 0.129 | 0.3256 | No |
| 33 | PRKAG2 | protein kinase AMP-activated non-catalytic subunit gamma 2 [Source:HGNC Symbol;Acc:HGNC:9386] | 4362 | 0.123 | 0.3261 | No |
| 34 | NUDT2 | nudix hydrolase 2 [Source:HGNC Symbol;Acc:HGNC:8049] | 4521 | 0.112 | 0.3192 | No |
| 35 | SLC25A12 | solute carrier family 25 member 12 [Source:HGNC Symbol;Acc:HGNC:10982] | 5506 | 0.032 | 0.2442 | No |
| 36 | ATP5PF | ATP synthase peripheral stalk subunit F6 [Source:HGNC Symbol;Acc:HGNC:847] | 6109 | -0.013 | 0.1979 | No |
| 37 | NME1 | NME/NM23 nucleoside diphosphate kinase 1 [Source:HGNC Symbol;Acc:HGNC:7849] | 6328 | -0.031 | 0.1825 | No |
| 38 | CAD | "carbamoyl-phosphate synthetase 2, aspartate transcarbamylase, and dihydroorotase [Source:HGNC Symbol;Acc:HGNC:1424]" | 6359 | -0.032 | 0.1817 | No |
| 39 | ATP5ME | ATP synthase membrane subunit e [Source:HGNC Symbol;Acc:HGNC:846] | 6365 | -0.032 | 0.1829 | No |
| 40 | ATP5F1E | ATP synthase F1 subunit epsilon [Source:HGNC Symbol;Acc:HGNC:838] | 6405 | -0.036 | 0.1816 | No |
| 41 | IL4 | interleukin 4 [Source:HGNC Symbol;Acc:HGNC:6014] | 6673 | -0.054 | 0.1634 | No |
| 42 | LDHC | lactate dehydrogenase C [Source:HGNC Symbol;Acc:HGNC:6544] | 7603 | -0.127 | 0.0973 | No |
| 43 | ATP6V1A | ATPase H+ transporting V1 subunit A [Source:HGNC Symbol;Acc:HGNC:851] | 8013 | -0.156 | 0.0731 | No |
| 44 | CTPS2 | CTP synthase 2 [Source:HGNC Symbol;Acc:HGNC:2520] | 8238 | -0.174 | 0.0642 | No |
| 45 | SLC25A13 | solute carrier family 25 member 13 [Source:HGNC Symbol;Acc:HGNC:10983] | 8713 | -0.210 | 0.0376 | No |
| 46 | FLCN | folliculin [Source:HGNC Symbol;Acc:HGNC:27310] | 8848 | -0.221 | 0.0380 | No |
| 47 | ENO1 | enolase 1 [Source:HGNC Symbol;Acc:HGNC:3350] | 9606 | -0.284 | -0.0071 | No |
| 48 | AK4 | adenylate kinase 4 [Source:HGNC Symbol;Acc:HGNC:363] | 9682 | -0.290 | 0.0013 | No |
| 49 | PKLR | pyruvate kinase L/R [Source:HGNC Symbol;Acc:HGNC:9020] | 10120 | -0.333 | -0.0164 | No |
| 50 | TAFAZZIN | "tafazzin, phospholipid-lysophospholipid transacylase [Source:HGNC Symbol;Acc:HGNC:11577]" | 10429 | -0.364 | -0.0226 | No |
| 51 | UCK2 | uridine-cytidine kinase 2 [Source:HGNC Symbol;Acc:HGNC:12562] | 10481 | -0.371 | -0.0084 | No |
| 52 | PPARGC1A | PPARG coactivator 1 alpha [Source:HGNC Symbol;Acc:HGNC:9237] | 10504 | -0.374 | 0.0082 | No |
| 53 | CTPS1 | CTP synthase 1 [Source:HGNC Symbol;Acc:HGNC:2519] | 10569 | -0.380 | 0.0219 | No |
| 54 | TMSB4X | thymosin beta 4 X-linked [Source:HGNC Symbol;Acc:HGNC:11881] | 10741 | -0.398 | 0.0281 | No |
| 55 | IMPDH1 | inosine monophosphate dehydrogenase 1 [Source:HGNC Symbol;Acc:HGNC:6052] | 11023 | -0.433 | 0.0274 | No |
| 56 | PPARA | peroxisome proliferator activated receptor alpha [Source:HGNC Symbol;Acc:HGNC:9232] | 11110 | -0.445 | 0.0425 | No |
| 57 | VPS9D1 | VPS9 domain containing 1 [Source:HGNC Symbol;Acc:HGNC:13526] | 11495 | -0.501 | 0.0371 | No |
| 58 | UCKL1 | uridine-cytidine kinase 1 like 1 [Source:HGNC Symbol;Acc:HGNC:15938] | 11515 | -0.503 | 0.0603 | No |
| 59 | MYC | "MYC proto-oncogene, bHLH transcription factor [Source:HGNC Symbol;Acc:HGNC:7553]" | 12801 | -0.970 | 0.0077 | No |
Table: GSEA details [plain text format]

  

Fig 2: GOBP\_RIBONUCLEOSIDE\_TRIPHOSPHATE\_BIOSYNTHETIC\_PROCESS      
 Blue-Pink O' Gram in the Space of the Analyzed GeneSet

  

Fig 3: GOBP\_RIBONUCLEOSIDE\_TRIPHOSPHATE\_BIOSYNTHETIC\_PROCESS: Random ES distribution      
 Gene set null distribution of ES for **GOBP\_RIBONUCLEOSIDE\_TRIPHOSPHATE\_BIOSYNTHETIC\_PROCESS**
